# Comparison of the Robustness of Pellet Film Coating with and without In-Process Coating Thickness Evaluation

**DOI:** 10.3390/pharmaceutics14112274

**Published:** 2022-10-24

**Authors:** Teja Brezovar, Sandi Svetič, Rok Dreu

**Affiliations:** 1 Krka d.d., Novo mesto, Šmarješka cesta 6, 1000 Ljubljana, Slovenia; 2 Faculty of Pharmacy, University of Ljubljana, Aškerčeva Cesta 7, 1000 Ljubljana, Slovenia

**Keywords:** pellets, coating of pellets, prolonged release coating, PAT, image analysis, residual water

## Abstract

The robustness of the pellet coating process with and without the use of an in-process coating thickness analyzer (PATVIS APA) was investigated. Pellets containing model drug were coated with a prolonged release film coating, using different process conditions. In the first set of experiments film coating was performed as process repetitions with unintentional variation of process parameters, and in the second set, controlled changes (inlet air humidity, gap between distribution plate and Wurster partition, starting pellet load) were made. Within the first set of experiments, the coating process endpoint was determined either via gravimetric consumption of coating dispersion or by means of in-line coating thickness monitoring. The release profiles of the pellets were analyzed and the density of coating calculated. Both methods of the process endpoint determination can be relatively robust in batch processing, if key factors influencing drug release profile are under control. PATVIS APA was shown to be a useful tool to better understand the coating process and can be helpful if coating process interruptions are encountered. Water content was shown to be the key factor influencing the drug profile, presumably by influencing the structure and thickness of the coating applied.

## 1. Introduction

Pellet coating is widely used process in pharmaceutical industry, especially for the production of sustained release dosage forms. Prolonged drug release ensures better patient compliance and less variation in plasma drug concentration profiles, while pellets allow for easier application and less inter- and intra-variability of drug absorption. The drug release rate through the film coating can be modelled after the Noyes—Whitney equation [1] and is defined by its thickness (*h* [m]), permeability (related to the permeability constant *K* [m^2^∙s^−1^)]), the total surface of the pellet (*S* [m^2^]) from which the release takes place and the concentration gradient ∆*c* [m^2^∙s^−1^], which is defined by the drug saturation concentration $c_{s}$ established within the reservoir system and by the drug concentration $c_{t}$ in the bulk fluid, as well as by local drug diffusion on both sides of the polymer membrane (Equation (1)).

(1) $$\frac{dm}{dt}=\frac{S}{h}\cdot K\cdot \left(\Delta c\right)\;\;\;\left[\mathrm{kg}\cdot s^{-1}\right]\;\;\;$$

The thickness of the prolonged release coating is a key parameter for achieving the targeted release profile as it directly affects the release of the active ingredient from the pellets [2,3,4]. As the thickness of the coating increases, the release rate slows down [5].

Traditionally, manufacturing processes, including the pellet coating process, are controlled by using validated and repeatable procedures and by controlling the incoming material properties, i.e., the total surface of the core material in combination with a fixed amount of coating suspension in the case of the pellet coating process. Knowing the total initial material surface allows us to calculate the applied dry matter in order to meet the planned thickness of the coating. Product quality is tested via coated sample analysis after the coating process is finished and adjustments to the coating can no longer be made [6]. In the case of coating process deviations, such as spray drying or agglomeration, the product quality may be insufficient, which is unfortunately shown only via the final off-line product analysis. Due to the out of specification result, e.g., too quick release profile, the batch of pellets is not to be used for further phases and released on the market [7,8].

A contemporary technological approach to control the process is implementation of process analytical technology (PAT). PAT is defined as a system for planning, analyzing and controlling technological processes by measuring critical process parameters, incoming materials and intermediate attributes when the process is still taking place. Tools are divided into 4 groups: multivariate tools for development, data collection and analysis, process analyzers, process management tools and tools for continuous improvement and knowledge management [4]. These tools allow monitoring and adjusting of process and product parameters during the process, thus integrating quality [9].

Process analyzers have improved significantly in recent years due to increased awareness of the importance of data collecting. Captured data are the basis for the real-time process control and can be combined with tools for multivariate data analysis [4]. Process analyzers can be at-line (sample is taken from the process and analyzed in time with analysis taking place near the process), on-line (the sample is taken from the process, but returned for further processing after completion of real-time analysis) and in-line (real-time analysis takes place within the process).

For control of pellet coating, many different process analyzers using different techniques are generally applicable. Most common are the spectroscopic techniques NIR and Raman spectroscopy, which are both non-destructive, rapid techniques that provide information about many sample properties simultaneously. Both, on the other hand, need complex calibration prior to their use [6,10,11]. The specificity of the NIR method is its sensitivity to moisture due to high absorbance of water, which is advantageous for water monitoring, but not when evaluating other attributes [12]. The terahertz pulsed imaging technique is ideal for characterization of crystalline properties such as polymorph quantification and detection of phase transitions [13,14], having the advantage of avoiding complex calibration and measuring interfaces located up to 3 mm below the surface [15]. One-dimensional optical coherence tomography (OCT) is also a non-destructive and contact free approach which provides in-depth B scan images of sample coating in real time and gives additional information about inter-batch variability [16].

Particle size and its distribution are critical quality parameters in many different pharmaceutical processes, and consequently, differential particle sizing techniques are an important group of PAT analyzers [17]. Coating thickness can be easily calculated through increases in particle size, and pellets with their defined shape and smooth surface are especially appropriate for such evaluations. Image analysis¸approach is apart from chord length distribution (CLD) evaluation via the focused beam reflectance measurement (FBRM) and spatial filtering velocimetry (SFT) [17,18], one of the most used particle sizing techniques in pharmaceutical manufacturing these days [9]. Particle size analysis techniques are based on measuring the size of the particles being coated, where the thickness of the coating is defined as the change in particle size. The advantage of gaining particle size data is also gaining information about a potential, unwanted agglomeration process. In addition, image analyses techniques also provide information about particle shape which cannot be assessed using other coating process analyzers [9,19]. In order to assure adequate measurements, it is necessary to coat the particles with a rather narrow, monomodal size distribution and to evaluate the appropriate particle sample size in a relatively short time span [7].

In early implementations of evaluating particle size distributions via image analysis, the assessment was performed in a static environment, using a digital camera [20,21] coupled with an optical microscope, while the first use of a dynamic image analysis approach was made in 2004 by Heinicke and Scwartz. They were able to show coating thickness differences of 4 µm for pellets ranging in size from 425 µm to 1400 µm and proved that image analyses algorithms can outline individual pellets and measure changes in dimensions of moving pellets efficiently within the off-line setup [22]. A further step in real-time monitoring of the coating process was made by Možina et al. [19] and Kadunc [23], where coating thickness and inter-pellet coating uniformity was measured in in-line mode through the observation window of the Wurster coater and the accuracy of the obtained results was proven via the reference spectroscopic method. The commercially available PATVIS APA system (Sensum d.o.o., Ljubljana, Slovenia), which performs real-time image analysis of pellets during the coating process [23,24], was developed on this principle and used in our study.

Although a few studies were made, their number is insufficient and additional work needs to be done to critically evaluate the value of the dynamic image analysis approach for the monitoring and control of the pellet coating process. The primary aim of the presented study was to compare the robustness of a realistic pellet film coating process end-point determination with and without in-process coating thickness evaluation using image analysis tool PATVIS APA, while keeping process conditions as constant as possible and by deliberately modifying them to estimate their effect on the drug release profile. A further aim of the study was to elucidate which process conditions affect film properties and their drug release performance the most and whether a dynamic image analysis tool can still be reliably used for film coating process endpoint determination under a broader range of process conditions. A drug layering process preceding the film coating experiments was also varied in scale and equipment in order to capture its possible influence on the final prolonged drug release profiles.

## 2. Materials and Methods

Neutral pellets 700–810 µm (Sugar spheres, Hanns G. Werner GmbH, Tornesch, Germany) were coated in two separate coating steps. For a layering process, water suspension composed of 15.9% *w*/*w* model drug in a form of weak acid salt, 1.9% *w*/*w* talc (Imerys Talc S.p.A., Porte, Italy) and 1.2% *w*/*w* hydroxypropyl cellulose (Ashland Specialty Ingredient g.p., NJ, USA) was used. For prolonged release, a coating combination of low and high permeable copolymer types of ethyl acrylate, methyl methacrylate and different levels of methacrylic acid ester with quaternary ammonium groups, i.e., Eudragits RS 30D (9.6% *w*/*w*) and RL 30 D (19.2% *w*/*w*) (Evonik Industries AG, Darmstad, Germany), with 1.7% *w*/*w* triethyl citrate (Vertellus LLC, Indianapolis, IN, USA) and 10.4% *w*/*w* of talc (Imerys Talc S.p.A., Porte, Italy) was used. All other materials used for dissolution tests were of analytical grade and were supplied commercially.

### 2.1. Preparation of Coated Pellets

#### 2.1.1. Manufacturing of Coated Pellets

The layering process was conducted in two separate processes, using different processing equipment. In coating experiments P1 to P3, each neutral pellet batch was drug layered separately using BX CGD1 (Brinox d.o.o., Medvode, Slovenia) lab scale coater, while for coating experiments P4 to P5 and all C experiments, one scale-up batch of pellets was layered using a pilot scale coater (MP 3/2/4-B2-F1/2, GEA Aeromatic, Wommelgem, Belgium).

Pellets were then film-coated in a controlled manner, using various process conditions to achieve prolonged release of the model drug. Film coating experiments were conducted in two different ways:

i. In P designated film coating experiments, a PATVIS APA in-process image-based analyzer was used for process monitoring and control, and the coating process was stopped when the target thickness of the film coating was achieved (experiments P1–P5, P-H (H for “humidity”), P-G (G for “gap”), P-Q (Q for “quantity”). We defined the target coating thickness in preliminary coating runs with the purpose of achieving near 90% model drug release in 6 h time. The target value of film thickness was determined via PATVIS APA as 9.4 µm.
ii. In C designated film coating experiments a classic manner of conducting the coating procedure was employed. The coating process was finished when the target mass of the suspension was sprayed onto pellets (experiments C1–C3 and C-H, C-G, C-Q). This quantity was defined in the specific preliminary experiment, where 463 g of suspension was sprayed and the coating thickness of 9.4 µm was achieved.

#### 2.1.2. Process Conditions Used in the Coating Experiments

(a) Without modification

Process parameters, that were kept approximately the same in all film coating experiments, were the mass of the starting drug-layered pellets (1000 g), equilibrium product temperature (28–30 °C) controlled by air flow (130 m^3^/h) and inlet air temperature (38–43 °C), spray rate (13.3–14.8 g/min) and atomizing air pressure (2.2 bar). In the P experiments (P1–P5), the mass suspension needed to achieve the target coating thickness (9.4 µm) was monitored, while in C designated experiments (C1–C3) the same amount (463 g) of suspension was always sprayed onto 1000 g of starting pellets. The binary nozzle with a tip diameter of 0.8 mm and cap opening diameter of 2.50 mm was used. After the spraying phase was finished, 10 min of drying within the fluid-bed chamber at an inlet air temperature of 38 °C was performed, and then, an additional curing step for 24 h at 40 °C in a drying chamber was performed.

(b) With modification

Three pairs of experiments were conducted with changed process parameters to assess which method of pellet processing (classic approach vs. using PAT tool) is more robust. For experiments P-H and C-H, the inlet air humidity was deliberately increased to 5.4–5.8 g/kg, while in all other experiments, it was kept in the range from 0.2 to 1.3 g/kg. By increasing the inlet air humidity, we wanted to increase the process yield as the process is consequently more moist in its equilibrium and the potential for spray drying is reduced.

For experiments P-G and C-G, the gap between the distribution plate and Wurster partition was reduced from 20 to 10 mm, and for experiments P-Q and C-Q, the quantity of the starting drug-layered pellet was lowered from 1000 to 600 g and the gap between the plate and partition was also reduced. The goal of the 2nd and 3th modifications was to lower the process yield by reducing the density number of the pellets within the draft tube and thus promote spray drying phenomena.

### 2.2. Characterization of Coating Process and of Coated Pellets

#### 2.2.1. In-Process Control of Coating Thickness Using Dynamic Image Analysis Tool

The process analytical technology visual inspection system for automated particle analysis PATVIS APA (Sensum d.o.o., Ljubljana, Slovenia) was placed on the chamber observation window so that pictures of pellets were taken in their down-falling phase of trajectory. We were continuously capturing 100 frames per second, using a short exposure time of 7 μs in order to avoid object blur. The resolution of the captured pictures was 848 × 848 pixels corresponding to 15.96 mm × 15.96 mm real image size. A calibrated telecentric lens optic with significant field depth was used to maintain object size independently of its distance from the observation window. The presentation of the results was performed once per minute in the form of coating thickness, calculated based on the difference between the median particle size for all captured and analyzed particles within 1 min time frame (typically 25,000 to 50,000 particles were included in the moving pellet size population) and a starting pellet core size median value from the reference time point at the beginning of coating. As a reference point, we defined the lowest value of particle size by measuring their D_50_, which was obtained in the initial minutes of coating, due to attrition, overdrying and shrinkage of the starting pellet cores. The standard error of the coating thickness was calculated as the half of the square root value of the sum of squared standard errors of means from the reference and selected time points [23]. In order to maintain sufficient visibility of the observation glass window, as the main technique challenge, a special pneumatic, magnetic driven wiper system with sharp scrape edges was used periodically throughout the processing time [24].

#### 2.2.2. Drug Assay and Dissolution Testing

Assay was determined on 2 parallels for each sample using approximately 300 mg of coated pellets. A sample was placed in a 100 mL volumetric flask, and 70 mL of medium (prepared by mixing 100 mL 0.1 M NaOH and methanol to 1000 mL mark) was added. After pellets disintegrated using an ultrasonic bath for approximately 15 min, medium was added to the flask marking. Then, 10 mL of solution was centrifuged (4000 rpm), and 3.0 mL of clear solution was diluted to 200 mL. UV-visible spectrophotometry was used to determine sample model drug concentration at a wavelength of 281 nm.

Dissolution testing with a pH change approach was used to determine the release kinetics of the model drug from pellets and to analyze resulting dissolution profiles in terms of their similarity, with the goal of verifying comparability of individual film-coated pellet batches. Dissolution testing was performed using approximately 260 mg of coated pellets per sample, which corresponds to 100 mg of drug, while each dissolution test consisted of 3 parallels. The rotating basket dissolution method was used during testing (100 rpm, baskets height 25 ± 2 mm, temperature of 37 °C). The initial medium for the first 2 h consisted of 750 mL of 0.1 M HCl with pH 1.2 (dissolution sample taken after 2 h); then, the second medium was established by adding 250 mL of 0.2 M Na_3_PO_4_·12H_2_O to raise the pH to 6.8. Dissolution samples were taken after 3, 4, 5 and 6 h. UV-visible spectrophotometry was used to determine the sample model drug concentration at a wavelength of 281 nm.

To numerically evaluate the similarity between two release profiles (1 and 2), we calculated similarity f_2_ factors using Equation (2), where n represents the number of dissolution profile time points, Rt is the % of drug released for profile 1 at time point t and Tt is the % of released drug for profile 2 at time point t.

(2) $$f_{2}=50\times log\left(\frac{1}{\sqrt{1+\frac{1}{n}\times \sum_{t=1}^{n}{\left(R_{t}-T_{t}\right)}^{2}}}\times 100\right)\;$$

Two drug release profiles are deemed similar when *f_2_* is between 50 and 100 [25].

#### 2.2.3. Loss on Drying (LOD) Measurements

To assess pellet sample water content, loss on drying (LOD) measurements on 5 g samples were performed using Mettler Toledo HR83-P (Mettler Toledo, Columbus, OH, USA) apparatus and employing a 20 min, 85 °C heating program. The change of the mass sample before and after the drying was expressed as sample percentage mass loss.

#### 2.2.4. Process Yield Calculation

Coating process yield was calculated using Equation (3):

(3) $$\gamma =\frac{m_{f}\times \left(1-LOD_{f}\right)-m_{s}\times \left(1-LOD_{s}\right)}{m_{susp}\times 0.207}\;$$

where *m_f_* and *m*_s_ are final and starting masses of pellets in the coater, 0.207 is the amount of dry matter in the suspension, *m_susp_* is the amount of sprayed suspension and *LOD_f_* and *LOD_s_* are the starting and final product LOD values.

#### 2.2.5. Pellet Size Measurements and Calculation of Specific Surface Area (SSA_V_, SSA_m_) and Pellets Densities

To determine the specific surface area (SSA) of different drug-coated pellets, the ferret diameter and CED (circle equivalent diameter) of each pellet (within a sample of known mass) were optically determined using a two-step approach assuming pellets were of spheroid shape. In the first step, we took pictures of pellets using a scanner (V700, Epson, Suwa-shi, Japan) with an image capturing resolution of 1200 dpi. In the second step, images were analyzed for the maximum and perpendicular ferret diameter using an image analysis program (based on open source machine vision library open CV 3.0). By knowing the dimensions of each pellet within the sample and mass of the samples, we were able to calculate their volume and then specific surface per unit volume, *SSA_v_*, and per unit mass, *SSA_m_*, which were determined using Equations (4) and (5).

(4) $$SSA_{V}=\frac{\sum S_{i}}{\sum V_{i}}=\frac{\frac{4\cdot \pi \cdot r^{2}}{4\cdot \pi \cdot r^{3}}}{3}=\frac{3}{r}=\frac{6}{d}\;$$

(5) $$SSA_{m}=\frac{\sum S_{i}}{m}=\frac{SSA_{V}}{\rho }\;$$

where *S_i_* represents the surface and *V_i_* the volume of an individual pellet, *d* is the average diameter of a pellet within the sample and *ρ* represents the granular density of the coated pellet.

The granular density of a drug-coated pellet, *ρ*, was calculated using Equation (6):

(6) $$\rho =\frac{m}{N\cdot V_{1}}\;$$

where m represents the mass of the pellet sample, *V*_1_ average volume of individual pellets, and *N* number of pellets in the sample.

Density of film coating was calculated using Equation (7):

(7) $$\rho_{obl}=\frac{3\cdot m_{susp}\cdot \omega }{N_{m}\cdot m_{cores}\cdot 4\cdot \pi \cdot \left({\left(\sqrt{\frac{SSA_{m}}{N_{m}\cdot 4\cdot \pi }}+h\right)}^{3}-{\left(\sqrt{\frac{SSA_{m}}{N_{m}\cdot 4\cdot \pi }}\right)}^{3}\right)}\;$$

where *m_susp_* is the mass of suspension transferred to pellets, *ω* is the mass fraction of dry matter within the suspension (0.207), *N_m_* is the number of particles per unit mass, *h* is the coating thickness and *m_cores_* is the mass of drug-coated pellets used.

#### 2.2.6. Scanning Electron Microscopy

The cross-sectional area of the pellets was imaged using a scanning electron microscope (Carl Zeiss Microscopy GmbH, Jena, Germany) at an accelerating voltage of 1.00 kV and at 2000-, 2500- and 5000-times magnifications. Prior to analysis, pellets were stuck to the SEM holder by means of carbon bi-adhesive tape.

## 3. Results

### 3.1. Process Monitoring, LOD Measurement and Process Yield Results

Two types of process monitoring were performed: image analysis PAT tool PATVIS APA was used for P designated experiments and the calculated amount of suspension for C designated experiments. For modified coating processes with lettering Q, H and G the staring pellet load, the inlet humidity and the gap between the distribution plate and the Wurster partition were varied. For film coating experiments P1 to P4, lab equipment drug-coated batches of pellets were used (API pellets 1a–1c). For all other experiments, the same pilot scale batch of drug-coated pellets was used (API pellets 2). An overview of the varied parameters of the film coating experiments, inlet air humidity, outlet air relative humidity (RH) and temperature (Tout), coating process yields and LOD values before and after the coating process, demonstrating water content, are presented in Table 1.

### 3.2. Coating Thickness Results Obtained by In-Line PATVIS APA System

Using the PATVIS APA tool, we used real-time diagrams of the pellets’ coating thickness changing over time for each P designated film coating experiment. The reference point for thickness measurements was the lowest point of the D50 parameter as the most representative pellet size measure. It is assumed this phenomenon is a consequence of pellet attrition before applying a film coating and initial water gain and loss, resulting in size change of the pellets (increase and shrinkage) happening at the beginning of each coating experiment when the spraying nozzle is activated and the process is not yet in thermal and mass transfer equilibrium.

**Figure 1:**
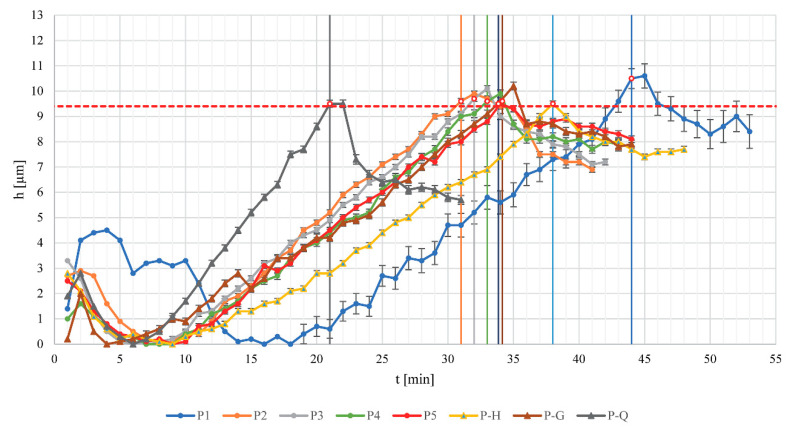
Pellet coating thickness measurement results presented through the coating time using PATVIS APA in-line PAT system; error bars represent estimated standard errors of coating thicknesses; vertical lines represent process endpoints determined via the manual process monitoring.

Apart from endpoint determination, using the PATVIS APA system during coating, we got insight into performed experiments. By changing the process parameters, different coating growth was achieved in a certain time (Figure 1), which shows the contribution of using the PAT tool. Coating was stopped, after the first (or second for P1) time point exceeded the previously determined target coating thickness of 9.4 µm. The coating thickness profile of the P1 experiment evidently showed some deviations from other similar processes (P2–P5) and is the consequence of starting process difficulties as the first experiment in the series. For modified processes P-H, we can observe the effect of the higher process humidity (Table 1) and the coating thickness increasing more slowly, presumably due to a higher coating density, as demonstrated later. For the P-Q experiment, although expressing the lowest coating yield (Table 1), due to a lower quantity of pellets in the process chamber the coating thickness grows faster as each particle is recirculating faster as the particle bed at the bottom of the chamber is shallower. Experiment P-G, with decreased gap size, demonstrated a similar coating thickness growth profile to those from the group of coating experiments P2 to P5.

### 3.3. Dissolution Testing and Drug Release Profile Comparison

**Figure 2:**
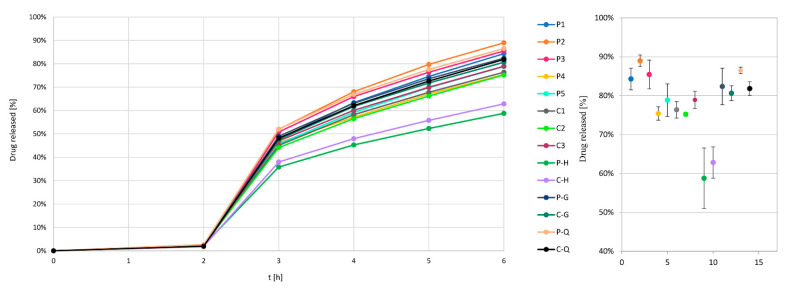
Drug release profiles in the first 6 h for all performed experiments (**left**), with separate presentation of % of released drug after 6 h (**right**).

**Figure A1:**
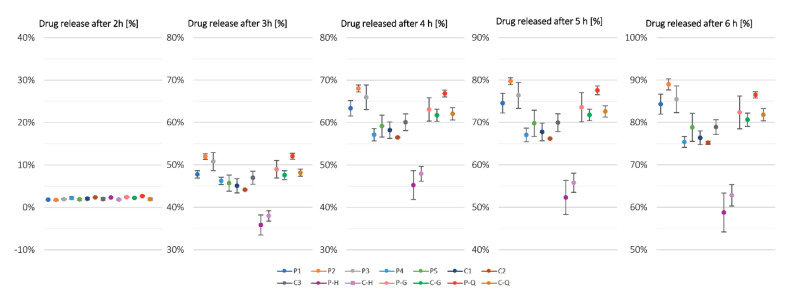
Drug release profiles in the first 6 h for all performed experiments represented as individual drug release data points with standard deviations depicted as error bars and organized as collections at time points 2 h, 3 h, 4 h, 5 h and 6 h.

Dissolution profiles presented in Figure 2 and in Figure A1 after 3 h evidently show four groups of dissolution results, i.e., (P1 to P3), (P4–P5 and C1–C3), (P/C-H), (P/C-Q and P/C-G). For easier evaluation and comparison, we focused on the amount of drug released after 6 h. Calculation of f2 similarity factors between individual release profiles, according to Equation (2), numerically confirms the existence of those four groups (Table 2).

Experiment groups P1 to P3 and P/C-Q and P/C-G have the fastest drug release profiles and high values of f_2_ factors (range 60–97, average 80). Film coating experiments conducted with the constant parameters, invariant of PAT system usage, where drug-loaded pellets stemmed from the same batch (API pellets 2), have lower profiles but are even more comparable (f_2_ range 74–96, average 85). Significantly lower drug release profiles were obtained for both experiments where inlet air humidity was deliberately increased (P-H and C-H). These profiles are mutually comparable (f_2_ = 76) but not comparable to practically all other profiles, with an f_2_ average of 42 (range 33–53).

## 4. Discussion

### 4.1. Evaluation of the Coating Outcomes for Experiments with Constant Parameters

The group of experiments conducted with constant process conditions are experiments P1 to P5 and C1 to C3. Based on release profile results, we can see that other factors seem to be more prominent for the drug release profile outcome, rather than usage of the in-line PAT analysis tool, which is based on the image analysis concept. By evaluating the differences in drug release profile between P1 to P3, P4 to P5 and C1 to C3 experiment groups, we see that for the first group (Table 1), each batch of neutral pellets was coated separately on a lab scale machine, and for the other experiments, the same batch of drug-loaded pellets was used as starting material. If we compare results of coating experiments with constant parameters P1 to P3 and C1 to C3, then we can conclude that the repeatability and hence the robustness of the coating experiments, all terms equal (same starting material and the same process parameters), do not change with implementation of the in-line PAT tool (RSD_6h,P_ = 2.8% vs. RSD_6h,C_ = 2.5%). However, it is worth mentioning that in the case of coating experiment P2, the weighing system failed during the coating experiment and this experiment was successfully finished solely on the basis of the PAT-determined process endpoint as the user had no information on the final dispersion consumption. This clearly demonstrates the merit of implementing a PAT system with the aim of monitoring the coating process.

### 4.2. Results of Off-Line Pellet Size Measurements and Calculation of SSA_m_ and Pellets Densities

With the aim of finding a reason for differences in drug release profiles between groups of experiments conducted at constant process parameter conditions, Ferret diameters of drug-loaded pellets, entering coating experiments, were measured in off-line mode using a scanner, and then, pellet granular density, SSAm and SSAv were calculated using Equation (4) through (6).

With the aim of understanding why drug release profiles were different for groups of pellets coated with API on a lab and on a pilot scale, we first calculated the SSAv and SSAm values for drug-loaded pellets entering the film coating process in both groups. Then, we verified whether identified differences in SSA values in combination with coating thicknesses should lead to different amounts of applied coatings to unify the release profile. We found out that we had already sprayed an appropriate amount of coating suspension on selected groups and that differences in SSA values between groups could not be the reason for differences in drug release profiles.

Pilot scale coated API pellets 2 were compared to lab coated API pellets 1a–1c, which were approximately 20 µm bigger, and the SSA_V_ was, as expected, lower (Table 3). This was in contrast with SSA_m_ results, which were, contrary to expectations, higher for pilot scale layered pellets, indicating the difference in the granular density of the pellets analyzed. Knowing that the release rate should actually be faster with a higher SSA_m_ and the same coating thickness, it was obvious that this was not the case for release profiles in reality. We assumed that there must be a difference in the permeability of applied SR coatings and that the difference must be somehow a consequence of incoming drug pellets as process parameter values were kept constant.

Trying to understand the difference between both groups, we calculated the correlation factors between process and material parameters and % of drug released after 6 h using Equation (8) (Table 4).

(8) $$K_{correlation}=\frac{\sum \left(x_{i}-\bar{x}\right)\cdot \left(y_{i}-\bar{y}\right)}{\sqrt{\sum {\left(x_{i}-\bar{x}\right)}^{2}\cdot \sum {\left(y_{i}-\bar{y}\right)}^{2}}}\;$$

Assuming strong correlation for factors with K_correlation_ > 0.9, we proved that residual water in the drug-loaded API pellets as starting material played a crucial role. API pilot scale coated pellets 2 have a much higher LOD value than API pellets 1 coated on a lab scale (Table 1). During the film coating process where pellets were exposed to drying conditions not resembling those in the layering process, the outgoing water acting as a general plasticizer must have influenced the properties of the formed coating. Expulsion of water from the pellet core, during the coating and curing process, can be substantiated by the ΔLOD values.

**Figure 3:**
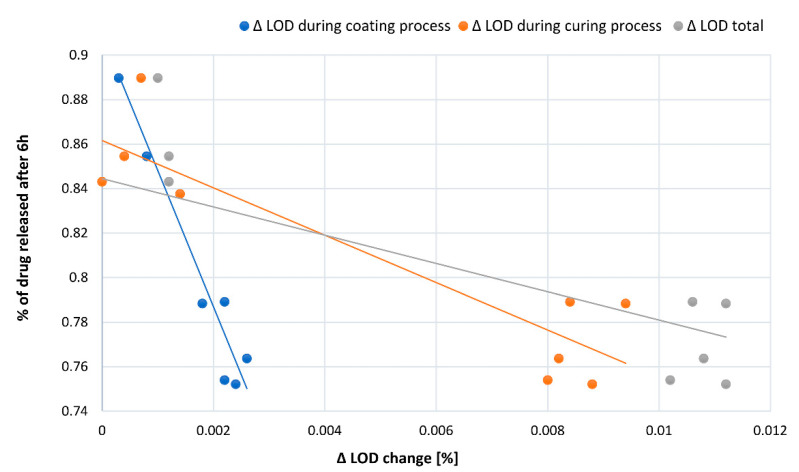
Correlation between drug released after 6 h and the change of pellets’ LOD during the coating and curing process for experiments with unmodified coating process P1–5 and C1–3.

Based on the correlation of Δ LOD during the process with the fraction of released substance after 6 h (Figure 3), we determined that observed profile changes between groups happened predominantly due to different changes in the moisture content of the pellets during the coating and curing process (Table 1). This is a consequence of different properties (water content) of incoming drug-loaded pellets. Knowing that water acts as a plasticizer [26,27], it is expected that water leaving the pellet core can significantly change film coalescence and its permeability.

**Figure 4:**
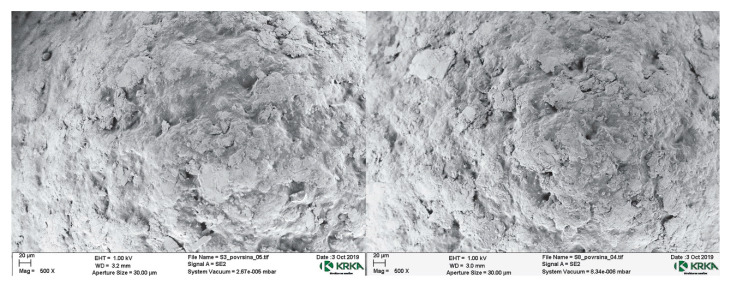
SEM micrographs of SR-coated pellets cross-sections: on the left the sample from the API pellets 1 group and on the right the sample from API pellets 2 group.

SEM micrographs of SR-coated pellets’ surfaces (Figure 4) revealed no significant differences in coating morphology between API pellets group 1 and API pellets group 2. Difference in coating structure at the micron level can therefore not be the underlying cause for differences in drug release profiles from both groups.

Based on all collected results we concluded that functional film coating with and without in-process control exhibits comparable robustness, if all process and product parameters are kept the same.

### 4.3. Evaluation of the Coating Outcomes for Experiments with Modified Parameters

In order to increase the process yield and, as was shown later, underline the importance of moisture as a general film plasticizer, the inlet air humidity (P-H, C-H) was deliberately increased. With the intention of decreasing the process yield, the amount of starting drug-layered pellets was reduced (P-Q and C-Q experiments; 700 g instead of 1000 g), and the gap between the partition and the distribution plate was also reduced in separate experiments (P-G and C-G; 10 mm instead of 20 mm). We assumed that PATVIS APA would help us to find the correct process endpoint and compensate for the change in process yield.

By raising the inlet air humidity, while maintaining the spraying rate, we slightly increased the yield of coating experiments P-H and C-H in comparison with other experiments (Table 1), and by decreasing the gap and pellet load, we managed to lower it, although this was not as repeatable as expected (P vs. C series, Table 1). The release profiles of pellets coated with and without in-process thickness evaluation for experiments P-H and C-H were comparable with each other (f_2_ = 76) but not with the profiles of previous samples (f_2_ = 33–53), which shows the great importance of water as a plasticizer in SR coating—the result matches the previous conclusions regarding differences between API pellets group 1 and 2 of the unmodified process. Contrary to expectations with regard to the expected and achieved higher process yield, when conducting experiment P-H with the PATVIS APA system, more coating dispersion was sprayed onto starting pellet cores then in the case of experiment without PAT system C-H (i.e., 513 g vs. 463 g). This can only be reasonable if the density of the coating was higher for coating experiments with increased inlet air humidity.

In the case of P-Q and C-Q experiments, practically the same amount of dispersion was sprayed on the pellet cores (279 g vs. 277 g), although in the case of P-Q, the coating yield was significantly lower in comparison with C-Q (62.2% vs. 70.8%), which resulted in a quicker drug release profile (86.5% vs. 81.8%). The PAT system should capture a decreased coating yield and prolong coating time, however, as it turns out, both LOD change before and after coating (Table 1) and core size reduction between the end of spraying and end of drying were highest in the case of the P-Q experiment in comparison with other coating experiments. This leads to an assumption that in the case of the experiment with fewer pellets, where individual particle circulation times reduce [28], drying during the spraying phase is not as efficient as in other experiments, thus leading to a premature process end when particle size is monitored during the coating experiment.

In order to better understand the drug release profile results, the density of the applied coating was estimated, using data of coating thickness after drying, measured with the PATVIS APA system and the pellet core SSA_m_ data obtained via the optical scanning method. Equation (7) was used for assessment of coating density (Table 5). Calculated coating densities are overestimated in their values as also pellet core is diminished in size during the drying stage. Only coating experiments with an equal initial pellet structure and LOD value can therefore be considered for comparison between experiments (i.e., PATVIS APA experiments with API pellets 2). Coating density is a good estimator of the coating morphology and most probably correlates inversely with the drug permeability coefficient through this membrane. 

Independently, we measured coating thickness using a He pycnometer (suspension was dried on a glass and real density of the formed film was measured on 2 parallels-) and got results of 1.7082 g/cm^3^ and 1.7055 g/cm^3^. These results prove that we have obtained realistic, however slightly elevated values, of the coating density through the calculation procedure.

**Figure 5:**
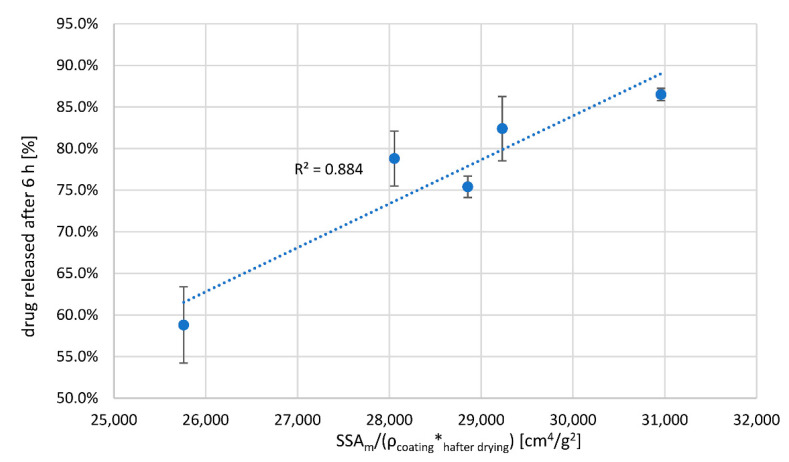
Correlation between percent drug released after and product of (SSAm/(ρ*h_PATVIS_)).

Knowing the density of the coating, its thickness and the SSA_m_ of pellets (Table 5), we considered the general Equation (1), while simplifying it by omitting the drug concentration gradient across polymer film, as for comparable core properties of the same formulation and sink dissolution conditions one can treat the drug concentration gradient across the polymer film as comparable between samples. The drug release rate was therefore described using measured coating thickness values, h, (higher h means lower release rate), measured SSA_m_ values (higher SSA_m_ increases release rate) and calculated coating densities, which were assumed to be in inverse correlation with drug permeability constant K. Three quantities were combined in a factor (SSA_m_/(ρ*h_PATVIS_)) and compared to percent drug released after 6 h (Figure 5). The correlation factor between percent drug released after 6 h and product (SSA_m_/(ρ*h)) is 0.94 when using measured coating thickness after drying phase. This demonstrates that the density of the coating matters as much as its thickness. Based on this finding, there is a need for development of in-line PAT analyzers that are able to concurrently track coating thickness as well as its density or structure. In this regard, the 1D OCT PAT analyzer fares better than simple dynamic image analysis systems, although the 1D OCT method also has its limitations, such as the use of pigments in coatings [29]. PAT analyzers based on dynamic image analysis can be implemented for coating process monitoring and control as long as critical process parameters that determine moisture equilibrium during coating and moisture content of the starting cores are tightly controlled.

## 5. Conclusions

Study results have demonstrated that both approaches of pellet coating process endpoint determination, either via an in-line dynamic image analysis system or via monitoring coating dispersion consumption, can be relatively robust in batch processing, if process parameters and starting core properties are tightly controlled. Nevertheless, PATVIS APA as an additional tool was shown to be very useful for better understanding of the coating process and can be helpful if coating process interruptions are encountered. It has been shown that understanding key material attributes and process parameters is crucial for the quality of the product. For the presented experiments and formulation, it was demonstrated that water content and its coating equilibrium are key factors influencing the prolonged drug release profile. Shortcomings of implementing simple coating thickness in-line PAT analyzers as process control have been demonstrated, if the coating process should be steered within a broader experimental space of process parameters and starting core critical properties. There is a clear need for the development of PAT analyzers that are capable of concurrent real-time coating thickness and structure-density evaluation.

## Figures and Tables

**Table 1:** Values of varied coating experiments parameters, of in- and outlet air moisture, LOD results of pellets before and after coating and process yields presented for individual film coating experiments.

Exp.	API Pellets	Pellets Load [g]	Gap [mm]	Moisture		LOD [%]			Process Yield [%]	Amount of Suspension [g]	Process End Point
				Inlet [g/kg]	Outlet RH [%] and T [°C] [%, °C]	Start	Finish	Δ			
P1	1_a	1000	20	1.3	22/29.5	0.72	0.60	0.12	62.6	453.0	Coating thickness
P2	1_b	1000	20	1.0	20/30.0	0.70	0.60	0.10	N/A	N/A	
P3	1_c	1000	20	0.6	20/29.6	0.72	0.60	0.12	67.9	459.0	
P4	2	1000	20	0.4	19/28.6	1.74	0.72	1.02	71.3	442.0	
P5	2	1000	20	0.2	18/28.7	1.78	0.66	1.12	71.3	453.3	
P-G	2	1000	10	0.2	19/27.8	1.80	0.70	1.10	66.4	457.0	
P-Q	2	600	10	0.2	18/28.7	1.82	0.54	1.28	62.2	279.0	
P-H	2	1000	20	5.8	34/29.1	1.78	0.66	1.12	74.8	513.2	
C1	2	1000	20	0.2	18/29.0	1.76	0.68	1.08	73.1	463.0	Amount of suspension
C2	2	1000	20	0.3	18/29.0	1.80	0.68	1.12	70.9	463.0	
C3	2	1000	20	0.3	18/28.6	1.80	0.74	1.06	69.7	463.0	
C-G	2	1000	10	0.2	19/29.0	1.79	0.68	1.11	71.6	463.0	
C-Q	2	600	10	0.5	19/28.5	1.82	0.56	1.26	70.8	277.0	
C-H	2	1000	20	5.4	34/29.5	1.78	0.64	1.14	74.7	463.0	

**Table 2:** Calculated similarity factors—f_2_ matrix for release profiles of conducted coating experiments; values below 50 are bolded; four distinctive groups from Figure 2—right are marked in different colors.

	P1	P2	P3	P4	P5	C1	C2	C3	P-H	C-H	P-G	C-G	P-Q	C-Q
**P1**	/	68	82	61	70	63	59	72	**37**	**41**	91	81	75	87
**P2**	68	/	79	50	55	51	**49**	56	**33**	**36**	65	60	86	63
**P3**	82	79	/	56	62	57	54	64	**35**	**39**	79	70	92	74
**P4**	61	50	56	/	81	94	92	79	**46**	**51**	64	70	53	67
**P5**	70	55	62	81	/	87	77	96	**42**	**47**	73	84	59	79
**C1**	63	51	57	94	87	/	90	83	**45**	50	66	73	55	70
**C2**	59	**49**	54	92	77	90	/	74	**47**	53	61	67	52	64
**C3**	72	56	64	79	96	83	74	/	**42**	**46**	77	89	60	82
**P-H**	**37**	**33**	**35**	**46**	**42**	**45**	**47**	**42**	/	76	**38**	**40**	**34**	**39**
**C-H**	**41**	**36**	**39**	51	**47**	50	53	**46**	76	/	**42**	**44**	**37**	**43**
**P-G**	91	65	79	64	73	66	61	77	**38**	**42**	/	88	73	95
**C-G**	81	60	70	70	84	73	67	89	**40**	**44**	88	/	66	95
**P-Q**	75	86	92	53	59	55	52	60	**34**	**37**	73	66	/	69
**C-Q**	87	63	74	67	79	70	64	82	**39**	**43**	95	95	69	/

**Table 3:** CED, SSAm, SSAv and density of API-coated starting pellets.

API coated pellets →	**1a**	**1b**	**1c**	**2**
SR pellets →	**P1**	**P2**	**P3**	**P4–P5, C1–C3**
median CED [µm]	1070.1	1078.6	1067.3	1094.2
SSA_m_ [µm^2^/mg] × 10^−6^	4.47	4.52	4.49	4.56
SSA_V_ [1/µm] × 10^3^	5.71	5.65	5.72	5.58
Density [g/cm^3^]	1.278	1.251	1.275	1.224
N/m [1/mg]	1.288	1.282	1.299	1.256

**Table 4:** Correlation factors between % of drug released after 6 h and process parameters and pellet properties.

Parameter	K_correlation_
Spraying time	−0.39
Spraying rate (average)	0.31
T _lab air_	−0.45
Humidity _lab_	**0.65**
Humidity _incoming air_	**0.72**
Humidity _outcoming air_	**0.58**
T _outcoming air_	**0.84**
T _pellets_	**0.60**
LOD API _pellets before coating_	**−0.93**
LOD SR _pellets before curing_	**−0.91**
LOD _pellets after curing_	**−0.82**
Δ LOD _during process_	**0.97**
Δ LOD _(API pellets to final pellets)_	**0.93**
Process yield	−0.41
CED _API pellets_	**−0.84**
h _end of spraying_	0.21
N _of pellets measured pellets using PATVIS APA_	0.38

**Table 5:** SSA_m_ for SR-coated pellets, coating thickness and density estimated after drying phase.

	P4	P5	P-G	P-Q	P-H
SSA_m_ [µm^2^/mg] × 10^6^	4.065	4.054	3.969	4.024	4.423
h _PATVIS-after drying_ [µm]	8	8.1	7.9	5.8	7.7
ƍ_coating_ [µm] (Equation (7))	1.76	1.78	1.72	2.24	2.23

## Data Availability

The data presented in this study are contained within the article.

## References

[1] Hattori Y., Haruna Y., Otsuka M. (2013). Dissolution process analysis using model-free Noyes–Whitney integral equation. Colloids Surf. B Biointerfaces.

[2] Tang E.S.K., Chan L.W., Heng P.W.S. (2005). Coating of Multiparticulates for Sustained Release. Am. J. Drug Deliv..

[3] Chauhan M.J., Patel S.A. (2012). A concise review on sustained drug delivery system and its opportunities. Am. J. PharmTech. Res..

[4] Guidance for Industry PAT—A Framework for Innovative Pharmaceutical Development, Manufacturing, and Quality Assurance. https://www.fda.gov/regulatory-information/search-fda-guidance-documents/pat-framework-innovative-pharmaceutical-development-manufacturing-and-quality-assurance.

[5] Akhgari A., Tavakol A. (2016). Prediction of Optimum Combination of Eudragit RS/Eudragit RL/Ethyl Cellulose Polymeric Free Films Based on Experimental Design for Using as a Coating System for Sustained Release Theophylline Pellets. Adv. Pharm. Bull..

[6] de Beer T., Burggraeve A., Fonteyne M., Saerens L., Remon J.P., Vervaet C. (2011). Near infrared and Raman spectroscopy for the in-process monitoring of pharmaceutical production processes. Int. J. Pharm..

[7] Knop K., Kleinebudde P. (2013). PAT-tools for process control in pharmaceutical film coating applications. Int. J. Pharm..

[8] Naelapää K., Veski P., Kristensen H.G., Rantanen J., Bertelsen P. (2010). Building quality into a coating process. Pharm. Dev. Technol..

[9] Korasa K., Vrečer F. (2018). Overview of PAT process analysers applicable in monitoring of film coating unit operations for manufacturing of solid oral dosage forms. Eur. J. Pharm. Sci..

[10] Jamrógiewicz M. (2012). Application of the near-infrared spectroscopy in the pharmaceutical technology. J. Pharm. Biomed. Anal..

[11] Kauffman J.F., Dellibovi M., Cunningham C.R. (2007). Raman spectroscopy of coated pharmaceutical tablets and physical models for multivariate calibration to tablet coating thickness. J. Pharm. Biomed. Anal..

[12] Hudovornik G., Korasa K., Vrecer F. (2015). A Study on the Applicability of In-line Measurement in the Monitoring of the Pellet Coating Process. Eur. J. Pharm. Sci..

[13] Shen Y.-C. (2011). Terahertz pulsed spectroscopy and imaging for pharmaceutical applications: A review. Int. J. Pharm..

[14] Haaser M., Gordon K.C., Strachan C.J., Rades T. (2013). Terahertz pulsed imaging as an advanced characterisation tool for film coatings—A review. Int. J. Pharm..

[15] Zeitler J.A., Shen Y., Baker C., Taday P.F., Pepper M., Rades T. (2007). Analysis of Coating Structures and Interfaces in Solid Oral Dosage Forms by Three Dimensional Terahertz Pulsed Imaging. J. Pharm. Sci..

[16] Koller D.M., Hannesschläger G., Leitner M., Khinast J.G. (2011). Non-destructive analysis of tablet coatings with optical coherence tomography. Eur. J. Pharm. Sci..

[17] Silva A.F.T., Burggraeve A., Denon Q., Van Der Meeren P., Sandler N., Van Den Kerkhof T., Hellings M., Vervaet C., Remon J.P., Lopes J.A. (2013). Particle sizing measurements in pharmaceutical applications: Comparison of in-process methods versus off-line methods. Eur. J. Pharm. Biopharm..

[18] Huang J., Kaul G., Utz J., Hernandez P., Wong V., Bradley D., Nagi A., O’Grady D. (2010). A PAT Approach to Improve Process Understanding of High Shear Wet Granulation Through In-Line Particle Measurement Using FBRM C35. J. Pharm. Sci..

[19] Možina M., Tomaževič D., Leben S., Pernuš F., Likar B. (2010). Digital imaging as a process analytical technology tool for fluid-bed pellet coating process. Eur. J. Pharm. Sci..

[20] Kennedy J.P., Niebergall P.J. (1997). Preliminary Assessment of an Image Analysis Method for the Evaluation of Pharmaceutical Coatings. Pharm. Dev. Technol..

[21] Larsen C.C., Sonnergaard J.M., Bertelsen P., Holm P. (2003). Validation of an image analysis method for estimating coating thickness on pellets. Eur. J. Pharm. Sci..

[22] Heinicke G., Schwartz J.B. (2005). Particle Size Distributions of Inert Spheres and Pelletized Pharmaceutical Products by Image Analysis. Pharm. Dev. Technol..

[23] Kadunc N.O., Šibanc R., Dreu R., Likar B., Tomaževič D. (2014). In-line monitoring of pellet coating thickness growth by means of visual imaging. Int. J. Pharm..

[24] Podrekar G., Kitak D., Mehle A., Lavrič Z., Likar B., Tomaževič D., Dreu R. (2018). In-Line Film Coating Thickness Estimation of Minitablets in a Fluid-Bed Coating Equipment. AAPS PharmSciTech.

[25] Moore J.W., Flanner H.H. (1996). Mathematical Comparison of Dissolution Profiles. Pharm. Technol..

[26] Steward P.A., Hearn J., Wilkinson M.C. (2000). An overview of polymer latex film formation and properties. Adv. Colloid Interface Sci..

[27] Hudovornik G., Vrečer F. (2015). Impact of the curing parameters on drug release from Eudragit RS and RL 30D coated pellets: Design of experiments. J. Drug Deliv. Sci. Technol..

[28] Kitak D., Šibanc R., Dreu R. (2018). Evaluation of pellet cycle times in a Wurster chamber using a photoluminescence method. Chem. Eng. Res. Des..

[29] Sacher S., Wahl P., Weißensteiner M., Wolfgang M., Pokhilchuk Y., Looser B., Thies J., Raffa A., Khinast J.G. (2019). Shedding light on coatings: Real-time monitoring of coating quality at industrial scale. Int. J. Pharm..

